# Prevalence and determinants of adolescent idiopathic scoliosis from school screening in Xiaoshan District, Hangzhou, China

**DOI:** 10.3389/fpubh.2025.1595793

**Published:** 2025-06-18

**Authors:** Qingyu Tu, Wenbin Xu, Yuqi Feng, Zaowei Zhang, Wei Zhuang

**Affiliations:** Jiangnan Hospital Affiliated to Zhejiang Chinese Medical University, Hangzhou, China

**Keywords:** idiopathic scoliosis, epidemiology, screening, adolescents, prevalence

## Abstract

**Objective:**

This large-scale epidemiological study aimed to determine the prevalence and associated risk factors of Adolescent Idiopathic Scoliosis (AIS) through school-based screening in Xiaoshan District, Hangzhou, China.

**Methods:**

A prospective cross-sectional study was conducted from 2023 to 2024, involving a total of 172,127 students aged between 7 and 18 years. A two-phase screening protocol was implemented: Phase I included physical examinations (assessing shoulder asymmetry and spinal curvature) alongside the Adams Forward Bend Test (with an angle of trunk rotation [ATR] ≥ 5°), while Phase II confirmed diagnoses through radiographic evaluation (Cobb angle ≥10°). Multivariate logistic regression analysis was employed to evaluate demographic, postural, and lifestyle factors.

**Results:**

The overall prevalence of AIS was found to be 1.23%, with a significant gender disparity observed (female: 1.71% vs. male: 0.92%, *p* < 0.001). Among the initial cohort of 4,482 screen-positive cases, hospital confirmation was obtained for 422 individuals, identifying a total of 199 AIS patients (146 mild cases, 47 moderate cases, and one severe case). Key risk factors identified included female gender (odds ratio [OR] = 2.742), postural abnormalities such as kyphosis (OR = 5.741), enrollment in junior high school (OR = 0.414), prolonged sedentary behavior exceeding 8 h per day (OR = 0.231), and family history of scoliosis (OR = 0.467). Notably, the prone position test effectively reduced false-positive rates by approximately 70.3%. Twin studies indicated no significant concordance regarding AIS diagnosis among siblings (*p* = 0.16).

**Conclusion:**

This study establishes that the prevalence of AIS in Xiaoshan District is consistent with national data reporting an incidence rate of approximately 1.2%. It highlights specific susceptibility based on gender as well as modifiable lifestyle risks associated with this condition. The integrated screening protocol that combines postural assessment with the Adams test demonstrates clinical utility for early detection efforts in schools. These findings underscore the necessity for preventive strategies within educational settings that focus on promoting proper posture education and encouraging increased physical activity among students.

## 1 Introduction

Adolescent Idiopathic Scoliosis (AIS), typically between the ages of 10 and 16 years, is diagnosed according to the Cobb angle of the spine (generally ≥10°), based on full-length standing anteroposterior X-ray results. In addition, AIS patients commonly experience serious vertebral rotation (1). While mild cases may cause postural issues and back discomfort, severe scoliosis can lead to cardiopulmonary impairment, disability, and psychological distress (2). Current research increasingly recognizes that the emergence of AIS coincides with the psychologically sensitive period of adolescence. Consequently, a significant proportion of AIS patients do not initially experience physical discomfort or functional limitations but develop psychological disturbances due to postural asymmetry and social scrutiny. Lee et al. demonstrated that patients with AIS exhibit significantly diminished self-image, predisposing them to adverse mental health outcomes (3). Severe and progressive AIS causes substantial harm to patient health and quality of life, including cosmetic deformity, cardiopulmonary impairment, chronic pain, and psychological morbidity. Early screening and intervention can effectively mitigate these risks and improve clinical outcomes.

American Commission on Chronic Disease definited screening as “the presumptive identification of unrecognized disease or defects through tests, examinations, or other procedures that can be rapidly applied” (1). AIS screening fits this definition and enables early intervention, potentially reducing surgical needs and treatment burden.

The prevalence of AIS varies worldwide from 0.93 to 12%. The global prevalence of AIS ranges from 0.93 to 12%, with current guidelines recommending regular screening for adolescents aged 8–18 according to the 2016 AIS guidelines (1). Early screening can detect spinal deformities at an early stage and allows for noninvasive interventions (e.g., core training and bracing), preventing curve progression and reducing surgical rates. Importantly, the correction efficacy for scoliosis in adults is unsatisfactory compared to that in minors, which may be attributed to epiphyseal closure in adults (4).

Currently, early screening for AIS is performed in various regions of the world. A screening study conducted in Hong Kong found that Over the five-year observation period, the prevalence rate and sensitivity for spinal curves exceeding 20°demonstrated an annual increase of 0.23%, while the positive predictive value (PPV) exhibited a progressive annual decline of 1.71%. This inverse correlation between enhanced detection metrics and reduced predictive accuracy suggests that school-based scoliosis screening maintains sustained clinical utility in identifying AIS cases requiring clinical monitoring while simultaneously highlighting evolving challenges in diagnostic precision within expanding screening populations (5).

International screening programs have demonstrated the importance of early detection. For example, Denmark’s study revealed more severe cases in non-school-based screening systems, whereas Singapore institutionalized AIS screening as routine healthcare (5). In China, studies on the prevalence of spinal deformities have also been conducted. For instance, Zhang et al. reported an overall combined prevalence of spinal deformities in primary and secondary school students in mainland China to of 1.02% (6). Furthermore. In 2019, a stratified cluster sampling study of 45,547 students in the Zhejiang region identified an overall prevalence of AIS of 3.9% (7). This screening activity was conducted in Xiaoshan, a more developed region in eastern China that has not previously undertaken AIS screening initiatives. This marked the first instance of implementing a large-scale two-stage screening process in Eastern China, resulting in the largest sample size ever collected in this area. Based on the gathered information, a statistical analysis was performed to identify the factors associated with positive AIS. This analysis serves as a foundation for subsequent screening.

## 2 Methods

This prospective cross-sectional epidemiological study was conducted from April 2023 to December 2024 in Xiaoshan, Hangzhou, Zhejiang Province. The participants underwent AIS screening concurrently with annual physical examinations. Recruitment in the Xiaoshan region was inclusive, without distinction based on geographic, economic, or ethnic background.

**Figure 1:**
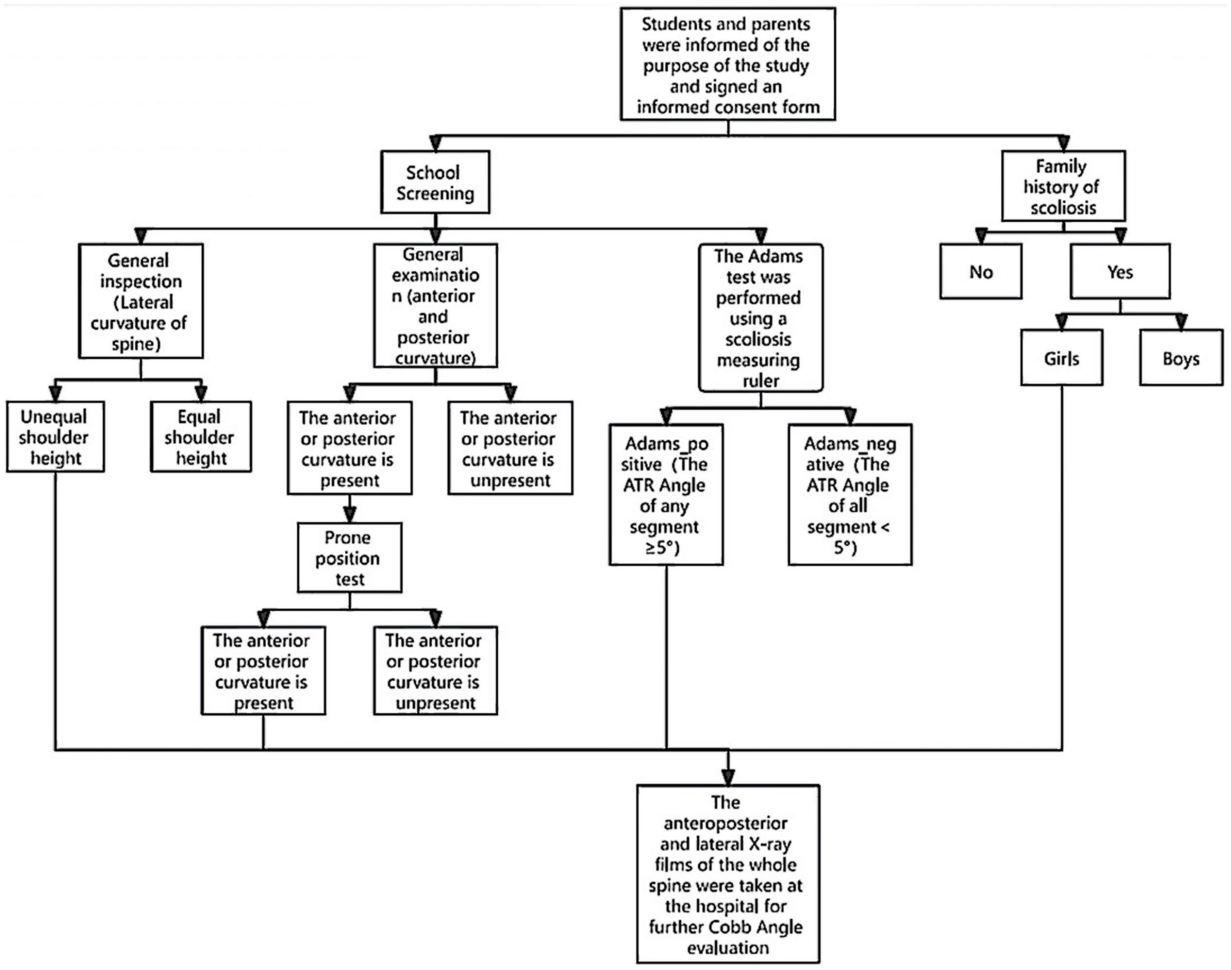
Flow chart of initial screening at the AIS campus. Abnormal posture on general examination, and positive Adams test, girls with a family history of scoliosis were recommended to go to hospital for the next stage of examination.

The study employed a two-step screening approach consisting of school-based screening (Phase I) and hospital diagnosis (Phase II), as depicted in Figure 1. The guidelines, regulations, and principles of the Declaration of Helsinki were strictly followed. Teachers, parents, and students were informed about the purpose of the study and the details of the examination. Informed consent was obtained from all participants and their parents, with signed consent forms.

**Figure 2:**
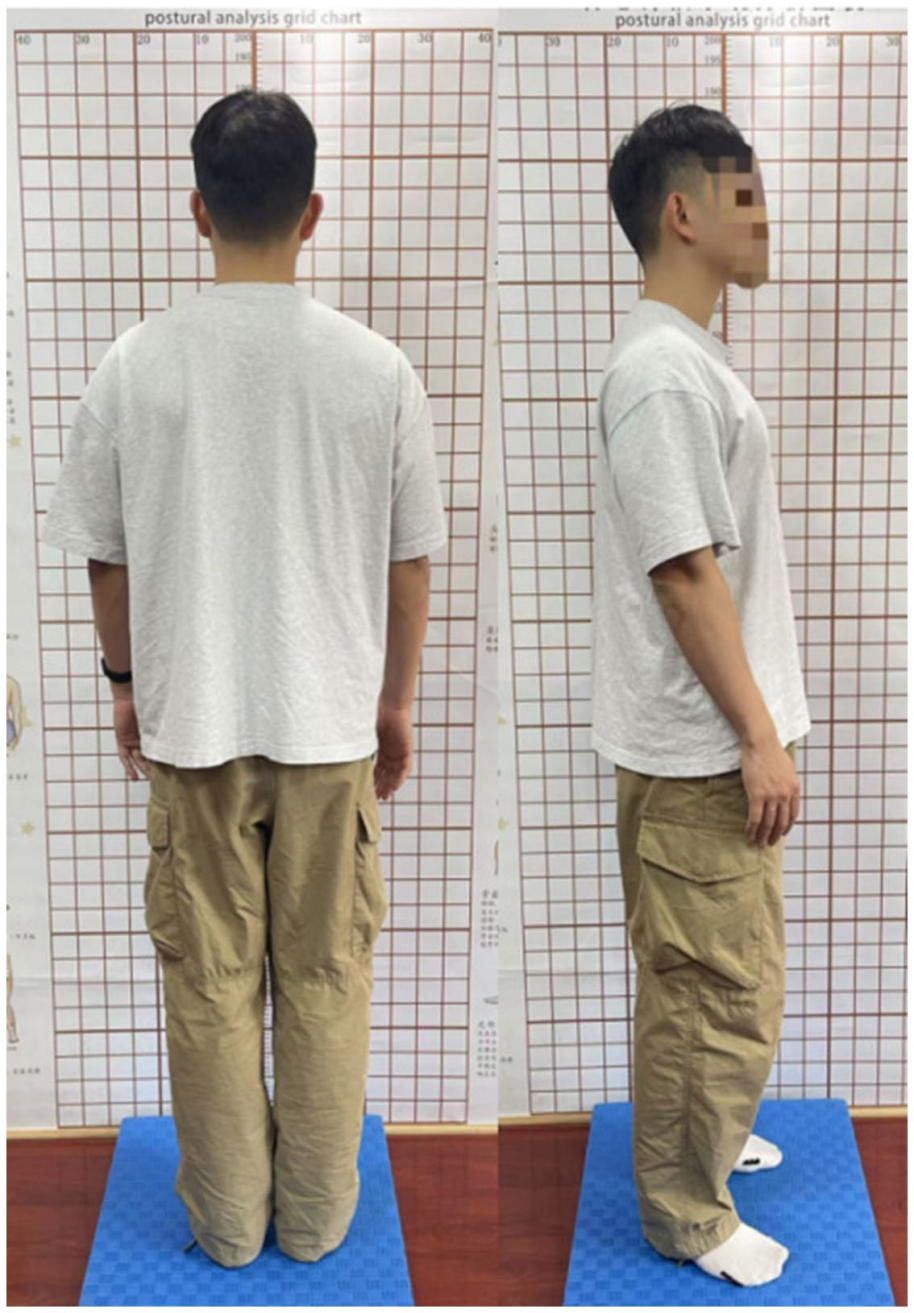
Standardized dorsal and lateral view photographs were obtained during the routine physical examination of the subject.

School-based screening was conducted by a team of pediatric orthopedic medical staff from the authors’ affiliated institutions and boys and girls were examined separately. During this phase, a physical examination was performed, which included checking for shoulder asymmetry, scapular prominence, waist or arm span inequality, and abnormalities involving the trunk or spine, such as humpback in the rib or lumbar region. Students with abnormalities at this stage were indicated to have anterior or posterior curvature present. The students were examined in a lateral standing position to observe whether the earlobe (external auditory canal), shoulder peak, and greater trochanter were aligned in a straight line (Figure 2). If the external auditory canal was behind the vertical line of the shoulder peak and greater trochanter, accompanied by significant anterior abdominal protrusion, increased posterior waist protrusion, and significant posterior protrusion of the buttocks, it was considered positive anterior convexity. If the external auditory canal was in front of the vertical line of the shoulder peak and greater trochanter, accompanied by a collapsed anterior chest, forward head and neck movement, and posterior indentation of the abdomen, it was considered a positive posterior convexity. Patients presenting with anterior and posterior convexity signs in the standing position were subjected to the prone test. The prone test required the examinee to lie flat on a hard board, relax, and the examiner observed whether the original anterior and posterior convexity signs still existed. This step helped distinguish between structural scoliosis and functional or postural scoliosis, as functional scoliosis may disappear after changing position, while structural scoliosis persists.

**Figure 3:**
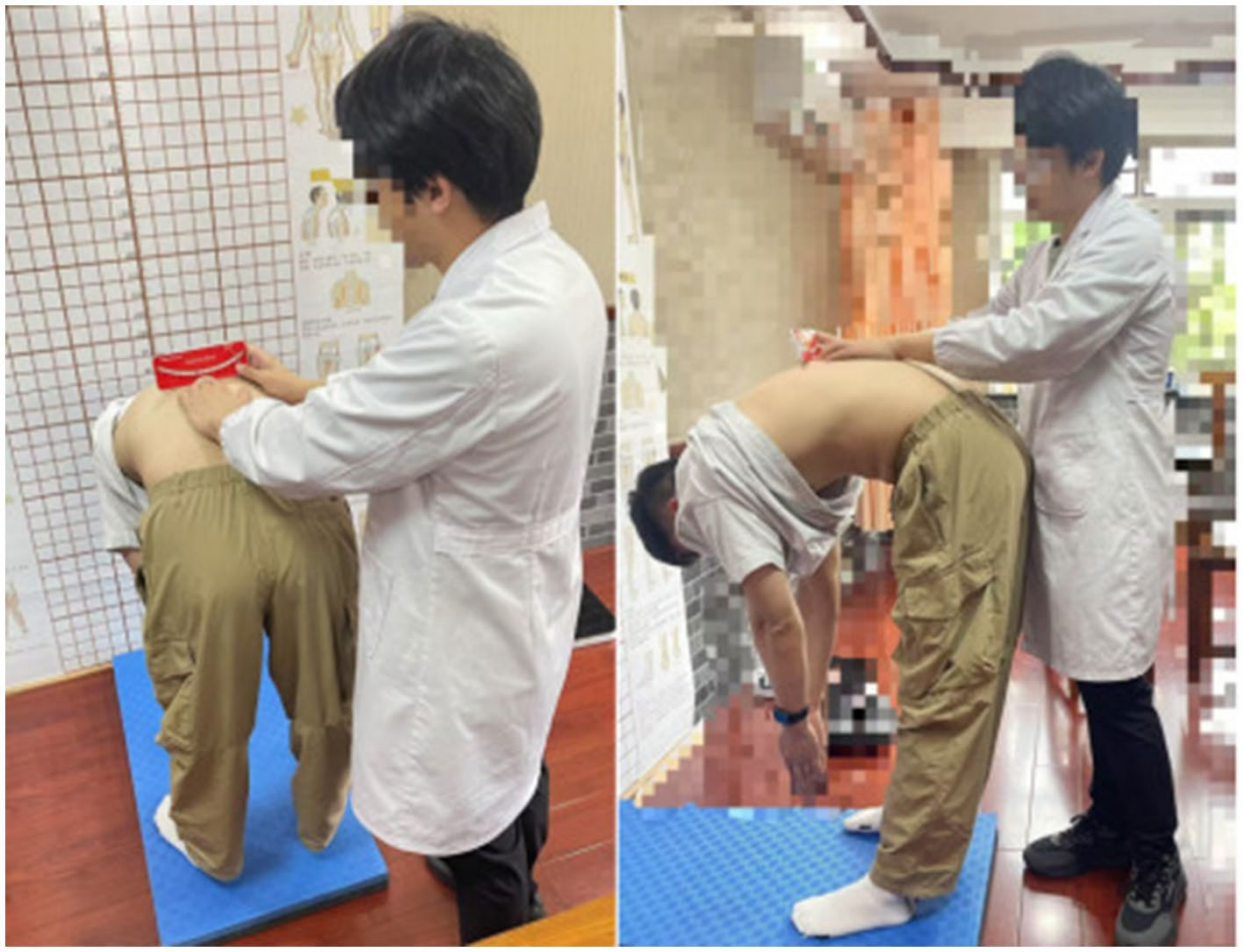
Standardized photographic documentation of both the subject in the Adams forward bending test position and the examiner's manual palpation procedure was obtained during the spinal deformity assessment.

Subsequently, the Adams Forward Bend Test (FBT) was performed. The child was exposed to the waist and back, maintaining an upright body position with feet together and legs straight, then bending forward 90°, allowing the upper limbs to naturally hang down, and placing the hands together between the knees. At this time, symmetry of the back shape was observed, and the spine on both sides of the back was checked from the thoracic vertebrae to the lumbar vertebrae with a spine measuring instrument to determine if they were of the same height. If the reading was less than 5°, spinal scoliosis was ruled out; otherwise, it was determined as spinal scoliosis (Figure 3). This process took approximately 1 min per student. Students with positive general examination tests and ATR angle readings of 5°or more in the thoracic, thoracolumbar, and lumbar segments, or with two other relevant factors (including a family history of scoliosis, being female, and abnormal standing or walking posture) were rescreened (8), and students with positive screening were transferred to hospital for full-length anteroposterior X-rays of the spine and further diagnosis and treatment.

Students diagnosed with AIS were advised to receive specific interventions from senior experts. Students who were positive in the school-based screening phase (ATR reading of ≥5°) but not diagnosed with AIS (Cobb angle of ≤ 9°) were followed up every 3–6 months.

During the school-based screening phase, basic information such as gender, age, and school attendance was collected; ATR readings and contact information (i.e., name, home address, and phone number) of students with positive screening were recorded, and data on posture signs, Cobb angle, and Risser sign level were collected after they participated in the hospital diagnosis. Students who participated in the hospital re-examination and were diagnosed with idiopathic scoliosis (Cobb angle ≥ 10°) were recorded as samples in the database. In this screening, 1.67% (*n* = 287) of the data were missing due to several factors, including some parents refusing to participate in the screening and not signing the informed consent form, as well as apparent transcription errors. The mechanism for missing data was confirmed to be random through Little’s MCAR test (*p* = 0.18). Given the low proportion of missing samples and the absence of systematic bias in their distribution, we employed complete case analysis (CCA) to exclude these missing samples, resulting in a total of 172,124 cases included in the analysis.

In this study, the core concept of propensity scoring is to provide reference data for AIS in the region through large-scale sample collection. Multicollinearity was evaluated by calculating Pearson correlation coefficients among the independent variables and variance inflation factors (VIF) derived from a multiple linear regression model that included the same dependent and independent variables. Tolerance values (1/VIF) were computed to assess the severity of collinearity. All VIFs were found to be below 5 (range: 1.00–3.12), and tolerance values exceeded 0.32, indicating no severe multicollinearity present in the data. The analytical protocol utilized a two-stage covariate selection methodology. Initial univariate screening eliminated confounding variables, retaining parameters demonstrating marginal associations (*p* < 0.2) for advanced multivariate analysis. These selected covariates were systematically integrated into a logistic regression model, controlling for intervariable interactions through variance inflation factor monitoring; this approach enabled precise identification of independent predictors while elucidating potential associations between positive clinical outcomes, thereby generating evidence-based insights for targeted screening initiatives. The statistical analysis for this study was conducted using the SPSS 24.0 software package. The processing of demographic data involved the use of frequency and descriptive statistics. In this study, a *p*-value less than 0.05 was considered statistically significant, with a confidence interval set at 95%.

## 3 Results

**Figure 4:**
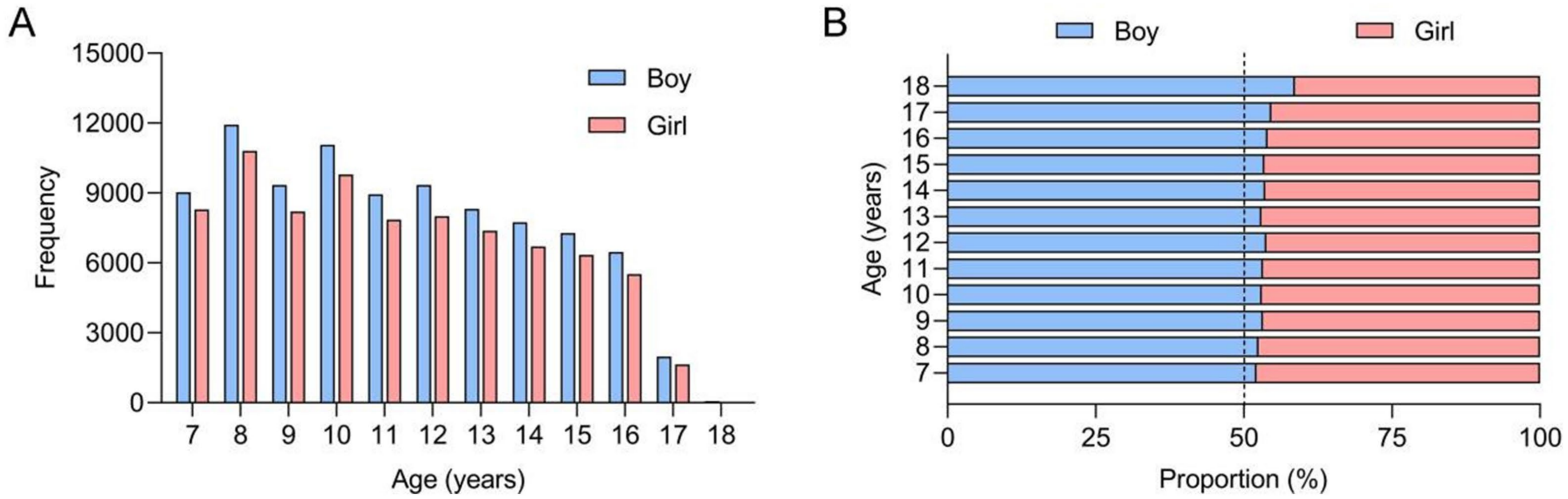
Histograms showing the frequency **(A)** and proportion **(B)** of the students participating in the school AIS screening in Xiaoshan District.

**Figure 5:**
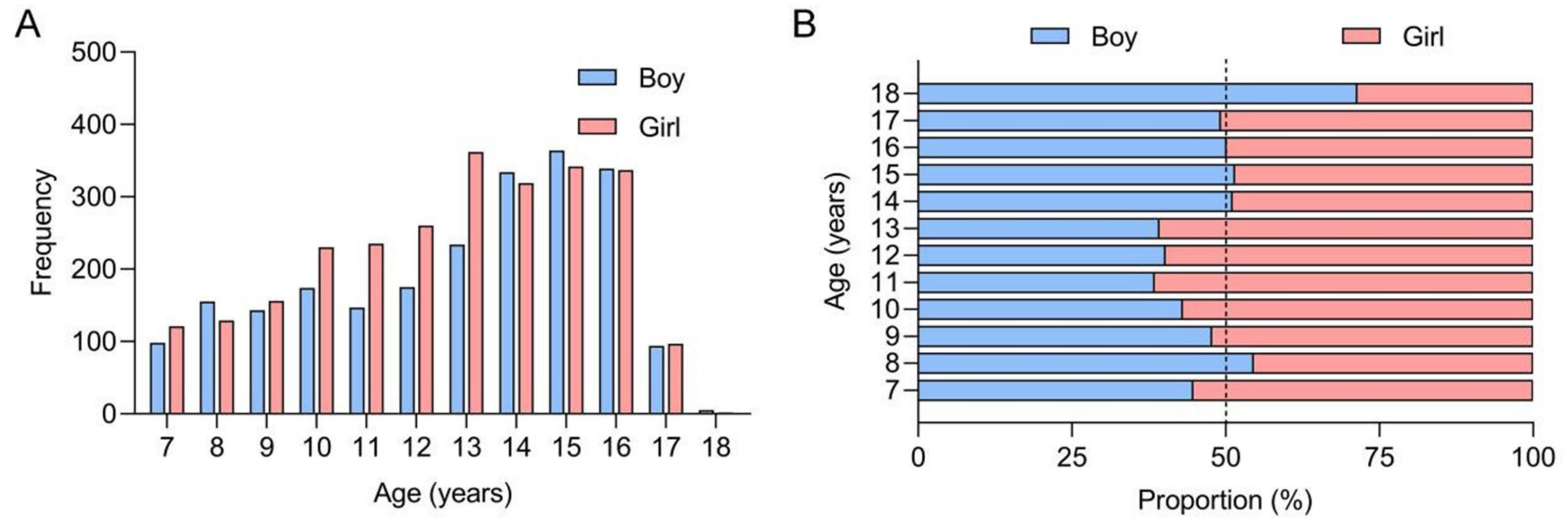
Histograms showing the frequency **(A)** and proportion **(B)** of the Adams-positive students participating in the school AIS screening in Xiaoshan District.

**Figure 6:**
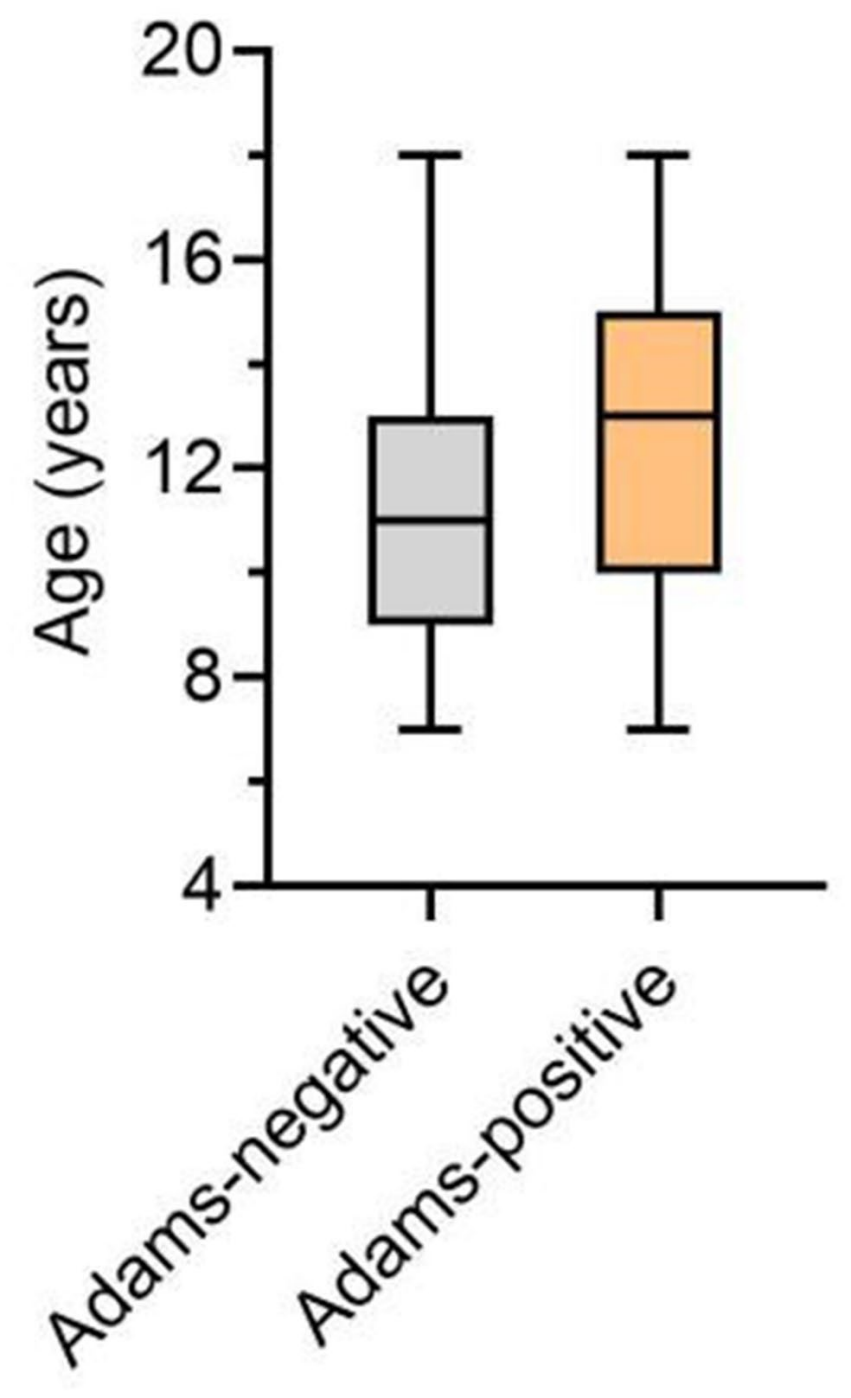
Box plot showing median (central dark line) quartiles 1 and 3, as well as minimum and maximum values as Adams-positive/negative groups for age.

A total of 172,127 primary and secondary school students (91,515 males and 80,609 females) participated in this screening study. The age and gender distribution of these children is illustrated in Figure 4. In the first phase of this school screening research, 4,482 adolescents (2,262 males and 2,590 females), representing 2.6% of the participants, were identified as positive cases requiring audiological evaluation; their age and gender distribution is shown in Figure 5. The box plot depicting the distribution of age among those who tested positive during initial screening can be found in Figure 6. Demographic information and related factors for these children are presented in Table 1.

**Table 1:** Demographic information and associated factors of children identified in the first phase of screening.

Variables	Total (*n* = 172,124)	Adams positive (*n* = 4,852)	*X* ^2^	*p*-value
Gender, *n* (%)			55.79	<0.001
Male	91,515 (53.2)	2,262 (46.6)		
Female	80,609 (46.8)	2,590 (53.4)		
Age, years, mean (SD)	11.21 (2.88)	12.7 (2.79)	-	<0.001
Unequal shoulders, *n* (%)	7,847 (4.6)	1701 (35.1)	10,673	<0.001
Anterior and posterior curvature, *n* (%)			1733.701	<0.001
Lordosis	80 (0.1)	14 (0.2)		
Kyphosis	353 (0.2)	79 (1.6)		
Period of study, *n* (%)			1724.116	<0.001
Primary school	117,783 (68.4)	2,162 (44.5)		
Junior high school	54,341 (31.6)	2,690 (55.5)		
Daily sitting time ≥8 h, *n* (%)	112,413 (65.3)	3,438 (70.9)	68.9	<0.001
Daily exercise time ≥1.5 h, *n* (%)	77,988 (45.3)	2093 (43.1)	5.556	0.018
Participate in dance activities, *n* (%)	18,462 (10.7)	640 (13.2)	31.92	<0.001
Family history, *n* (%)	3,615 (2.1)	162 (2.3)	36.77	<0.001

Among them, 422 individuals underwent further examination at Xiaoshan District Traditional Chinese Medicine Hospital, resulting in an enrollment rate of 9.4%. There were no statistically significant differences between the demographic characteristics—such as gender (*p* = 0.058) and age (*p* = 0.093)—of the enrolled group of 422 individuals and that of the initial positive cases totaling 4,482.

In a cohort of children undergoing radiological examination, 59 males and 140 females were diagnosed with AIS. The follow-up diagnosis rate 47.2%, with an overall prevalence of 1.23%. Notably, the prevalence among females (1.71%) was significantly higher than that among males (0.92%) (*p* < 0.001; Table 2).

**Table 2:** Demographic information of AIS and multivariate logistic regression analysis parameters.

Variables	Cobb angle (10°–19°)	Cobb angle (20°–40°)	Cobb angle (>40°)	OR	95% CI	*p*-value
Gender, *n*				2.742	2.019–3.726	<0.001
Male	99	38	1			
Female	47	9	0			
Unequal shoulders, *n*	36	15	0	0.136	0.098–0.189	<0.001
Anterior and posterior curvature, *n*				5.741	2.417–13.635	<0.001
Lordosis	1	0	0			
Kyphosis	4	1	0			
Period of study, *n*				0.414	0.312–0.549	<0.001
Primary school	73	14	1			
Junior high school	73	33	0			
Daily sitting time ≥8 h, *n*	42	16	1	0.231	0.170–0.313	<0.001
Daily exercise time ≥1.5 h, *n*	69	18	1	1.020	0.770–1.350	0.891
Participate in dance activities, *n*	19	6	0	1.271	0.540–1.923	0.256
Family history, *n*	49	13	0	0.467	0.345–0.631	<0.001

Utilizing a multivariate logistic regression model, we identified significant correlations between the prevalence of AIS and factors such as gender, shoulder alignment and kyphotic posture, educational stage, sedentary time, and family history of scoliosis.

In a recent campus screening conducted in the Xiaoshan area, 1,624 twins were assessed. Of these, 32 individuals initially screened positive for AIS. During follow-up screening, 8 pairs of twins were included (all 8 pairs of twins were identical twins). Among these, 3 pairs were simultaneously diagnosed with AIS, 1 pair had one confirmed twin, and the other was not confirmed. The McNemar test yielded a *p*-value of 0.16, indicating that there was no statistically significant difference between the concordance rate and discordance rate of AIS among twins.

Among the 199 patients with scoliosis included in this study, 146 exhibited mild AIS (10°–19°). For these patients, it is recommended that they undergo specialized rehabilitation training and engage in at least half an hour of daily rehabilitation exercises. Additionally, 47 patients were classified as having moderate AIS (20°–39°); for those with a Risser sign ≤2, it is advised that they wear braces for at least 21 h per day. Lastly, one patient presenting severe AIS (greater than or equal to 40°) has been recommended for surgical intervention.

## 4 Discussion

### 4.1 Gender influence on AIS

Our population-based screening of 7-18-year-old students in Xiaoshan District, Hangzhou revealed significant gender disparities in AIS prevalence. Female participants demonstrated higher AIS positive rates compared to males (*p* < 0.05), while shoulder asymmetry showed no significant gender difference (*p* = 0.776). Notably, post-prone position testing revealed a distinct pattern with males exhibiting higher true anteroposterior convexity prevalence (0.34% vs. 0.15%, *p* < 0.05), a phenomenon currently lacking definitive explanation in existing literature. Consistent with previous reports from Guangdong, Wuxi, and Shanghai, our findings confirm the established pattern of earlier onset and greater disease susceptibility in females, though Cobb angle measurements showed no significant gender-based variation (*p* = 0.352) (8–10).

Emerging mechanistic studies suggest two principal etiological factors in female predisposition: leptin signaling abnormalities and estrogen receptor gene polymorphisms. Experimental evidence from Wu et al. demonstrates elevated AIS incidence in leptin-overexpressing murine models (27). Clinical investigations by Liu et al. revealed altered leptin bioavailability in 95 AIS-affected girls compared to 46 controls, as measured through ELISA quantification of leptin and soluble leptin receptor (sOB-R) concentrations (12). Qiu et al. further established significant correlations between leptin levels and anthropometric parameters (age, menarche status, BMI) as well as bone mineral characteristics in AIS patients (11). Complementary genetic research by Inoue et al. identified estrogen receptor gene polymorphisms associated with curve severity progression, suggesting potential DNA-based prognostic markers (13). Wu et al.’s case–control study (202 AIS vs. 174 controls) specifically implicates XbaI polymorphism in estrogen receptor genes as a potential risk modifier (14).

### 4.2 Postural impact on AIS detection

Our screening protocol innovatively combined traditional physical examination with Adams forward bend test and prone position verification to minimize measurement errors. Contrary to previous screening paradigms that discounted general postural assessment, we identified significant correlations between clinical signs (shoulder asymmetry, anteroposterior convexity) and AIS diagnosis (*p* < 0.01), warranting their inclusion in primary screening criteria. While mild AIS (Cobb angle ≤20°) primarily manifests as cosmetic concerns with psychological implications, its visible postural markers demand clinical attention to address adolescents’ quality of life concerns.

The prone position test proved particularly effective in differentiating structural from postural deformities, eliminating 70.3% of false-positive anteroposterior convexity findings. This aligns with Brink et al.’s radiographic validation of prone positioning in assessing true spinal morphology across imaging modalities (15). He et al.’s comparative analysis of four positions (supine, prone, lateral bending sitting, lateral bending prone) further substantiates prone positioning’s utility in predicting initial orthotic treatment outcomes through spinal flexibility assessment (16).

### 4.3 Lifestyle factors in AIS pathogenesis

Multivariate logistic regression analysis identified prolonged sedentary behavior (>8 h/day) as significant risk factors (OR = 2.34, 95% CI 1.89–2.91). Our data reveal an inverse relationship between academic progression and physical activity levels, with primary school students demonstrating lower AIS prevalence (0.9%) compared to secondary counterparts (1.8%, *p* < 0.01). These findings corroborate Glavaš et al.’s Croatian cohort study highlighting increased AIS risk in sedentary, low-BMI adolescents (17). Notably, while asymmetric sports participation showed no association with AIS development, we advocate for school-based interventions to increase structured physical activity as primary prevention. Mechanism Underlying the Association between Prolonged Sedentary Behavior and Increased AIS Prevalence. Current evidence indicates that sustained indoor sedentary behavior in children may limit opportunities for sunlight exposure, thereby increasing the risk of vitamin D deficiency when compared to peers who engage in regular outdoor activities. Notably, Ng et al. identified a dual correlation pattern of 25-hydroxyvitamin D3: a positive association with bone mineral density (BMD) in healthy adolescents and an inverse correlation with Cobb angle severity in AIS patients. Collectively, these findings indicate that vitamin D deficiency may constitute a modifiable risk factor in AIS pathogenesis, potentially through its regulatory effects on skeletal mineralization and spinal biomechanical stability (18). The findings from the current investigation revealed no statistically significant associations between daily exercise duration (≥1.5 h per day) or participation in dance activities and the incidence of Adolescent Idiopathic Scoliosis (AIS). Methodological constraints in assessing exercise modalities limited our ability to delineate specific movement patterns among pediatric participants. This limitation is consistent with the meta-analysis conducted by Yue Peng et al., which identified physical activity as a contentious risk factor, suggesting that different movement paradigms and training protocols may lead to divergent pathophysiological outcomes (19). Notably, McMaster’s comparative study demonstrated a positive correlation between enhanced toe-touch flexibility and AIS prevalence (20). While Watanabe’s epidemiological survey indicated potential associations between classical ballet training and AIS development (21), current evidence remains inconclusive regarding other dance genres. The methodological framework of our study, which lacked detailed documentation of dance subtypes, consequently restricted analytical precision to examining broad categorical associations between dance participation and AIS risk.

### 4.4 Genetic contributions to AIS

Although family history emerged as a moderate risk factor (OR = 1.56), our twin study subset analysis (*n* = 32 pairs) failed to reach statistical significance, potentially limited by sample size and zygosity determination challenges. Current evidence suggests polygenic inheritance patterns, with Carr et al. reporting higher concordance rates in monozygotic (76%) versus dizygotic twins (32%) (22). Rhijin et al. conducted a systematic analysis of large-scale cohort data from studies on AIS and found that while twin pairs exhibited significant genetic concordance in disease susceptibility, the severity of spinal curvature and three-dimensional spatial vector characteristics demonstrated stronger statistical associations with postnatal environmental exposure factors (23). Prior epidemiological studies demonstrate that genetic predisposition to the condition manifests with varying prevalence rates among family members: 11% for first-degree relatives, 24% for second-degree relatives, and 1.4% for third-degree relatives (24). The complex interplay between genetic predisposition (estimated heritability 38–80%) and environmental modulators remains under investigation, with no single causative gene yet identified (25).

### 4.5 Conclusions and future directions

Our epidemiological survey established the AIS prevalence in Xiaoshan District was 1.23% (females: 1.71%, males: 0.92%), alignied with national meta-analytic estimates (1.2% in Chinese adolescents). The identified risk determinants included postural abnormalities, sedentary lifestyle, and familial predisposition. A recent meta-analysis revealed that the prevalence of AIS among Chinese adolescents aged 10–18 years was 1.2%, with a notable upward trend observed in recent years, particularly exhibiting marked increases between 2016 and 2024 (references pending) (26). A 2021 cross-sectional study on spinal curvature status and influencing factors among Zhejiang’s primary and secondary school students documented a significantly higher prevalence rate of 3.9% (references pending) (7). While the exact etiological mechanisms remain unclear, this increasing incidence pattern may be attributable to both intensified academic pressure and reduced physical activity levels among mainland Chinese students, as well as enhanced public health efforts, including large-scale proactive AIS screening programs and health education campaigns that have increased the clinical detection rates of asymptomatic cases.

The study limitations include incomplete follow-up data (9.4% hospital confirmation rate) and subjective activity reporting methodology. Future investigations should employ accelerometer-based activity monitoring and longitudinal design. The absence of pathognomonic genetic markers underscores the need for multi-omics approaches to elucidate AIS pathogenesis. Public health initiatives should prioritize school-based screening integration with ergonomic education to mitigate this growing pediatric orthopedic concern.

## Data Availability

The original contributions presented in the study are included in the article/Supplementary material, further inquiries can be directed to the corresponding author.
